# Supplementary material for: Characterization of Human Subcutaneous Adipose Tissue and Validation of the Banking Procedure for Autologous Transplantation
Source: Int J Mol Sci. 2023 May 3;24(9):8190. doi: 10.3390/ijms24098190 (PMC10179225; doi:10.3390/ijms24098190)
Supplement: Supplementary file 1 [file ijms-24-08190-s001.zip › ijms-2239320-supplementary.pdf]

Supplementary figures

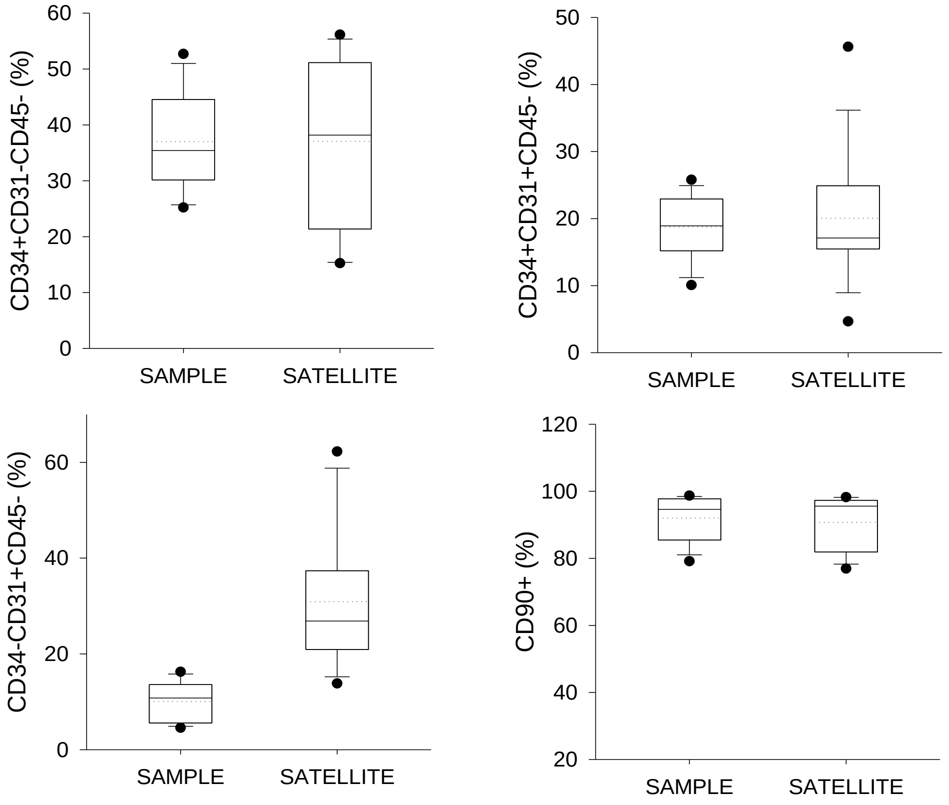

Figure S1. Immunophenotyping of the SVF (stromal vascular fraction) performed by flow cytometry in cryopreserved adipose tissue samples and their paired-quality controls (satellite). Plots showed the quantification of adipose stromal/stem cells (ASCs, CD34+CD31−CD45−), endothelial progenitor (CD34+CD31+CD45-) and endothelial mature (CD34-CD31+CD45-) cells contained in SVF. The percentages of each class are displayed as box plot graphs where 5th and 95th percentiles are highlighted by black circles, the medians by solid lines, and the means by dotted lines. The data were analyzed using one way ANOVA on Ranks.

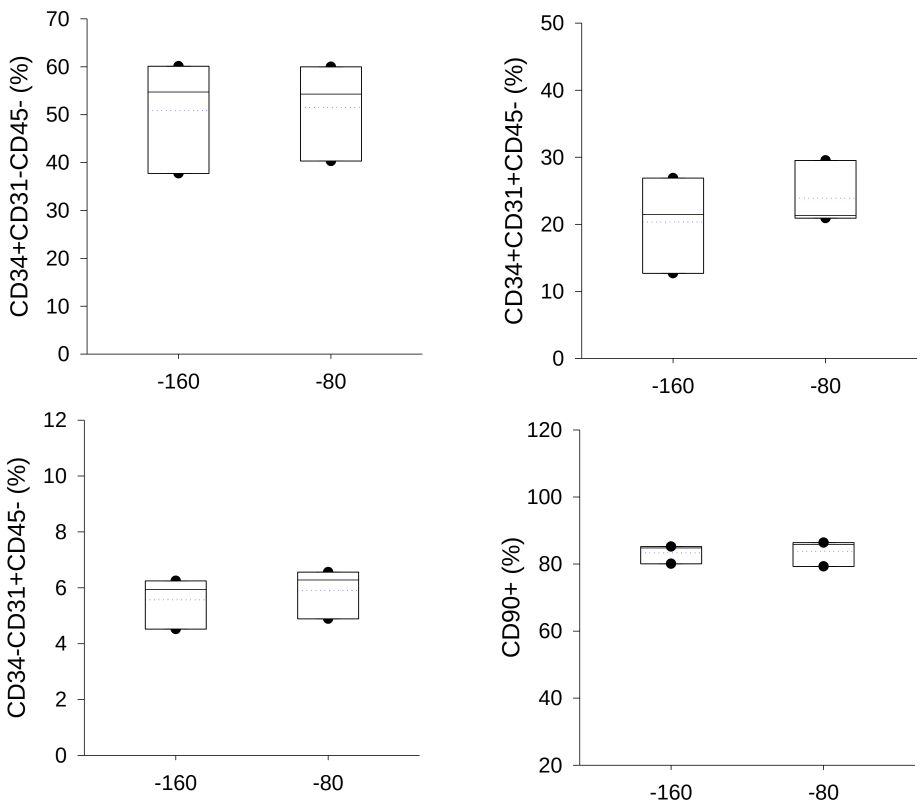

Figure S2. Immunophenotyping of the SVF (stromal vascular fraction) in cryopreserved (vapor phase liquid nitrogen, -160) and sample kept in dry ice for 24h (-80) by flow cytometry. Plots showed the quantification of adipose stromal/stem cells (ASCs, CD34+CD31−CD45−), endothelial progenitor (CD34+CD31+CD45-) and endothelial mature (CD34-CD31+CD45-) cells contained in SVF. The percentages of each class are displayed as box plot graphs where 5th and 95th percentiles are highlighted by black circles, the medians by solid lines, and the means by dotted lines. The data were analyzed using one way ANOVA on Ranks.

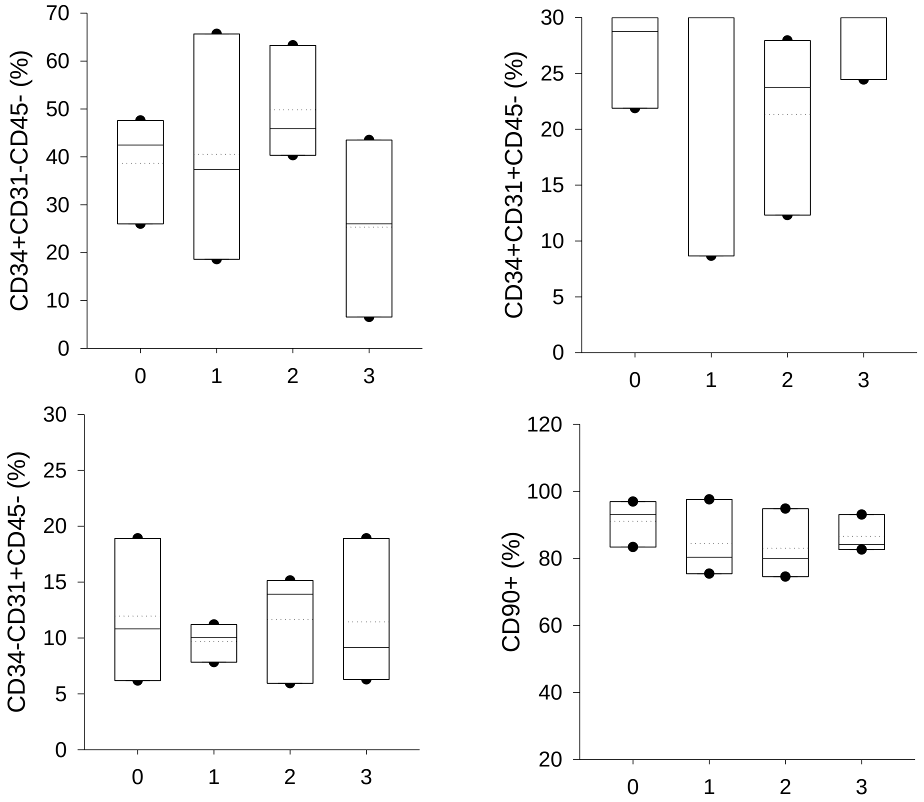

Figure S3. Immunophenotyping of the SVF (stromal vascular fraction) in cryopreserved samples (0) and samples kept at -80°C for a short-term storage (1-3: 1,2 and 3 month of storage) by flow cytometry. Plots showed the quantification of adipose stromal/stem cells (ASCs, CD34+CD31−CD45−), endothelial progenitor (CD34+CD31+CD45-) and endothelial mature (CD34-CD31+CD45-) cells contained in SVF. The percentages of each class are displayed as box plot graphs where 5th and 95th percentiles are highlighted by black circles, the medians by solid lines, and the means by dotted lines. The data were analyzed using one way ANOVA on Ranks.

0: cryopreserved lipoaspirate, 1: 1-month storage, 2: 2-months storage, t3: 3-months storage. The storage was performed at -80°C.

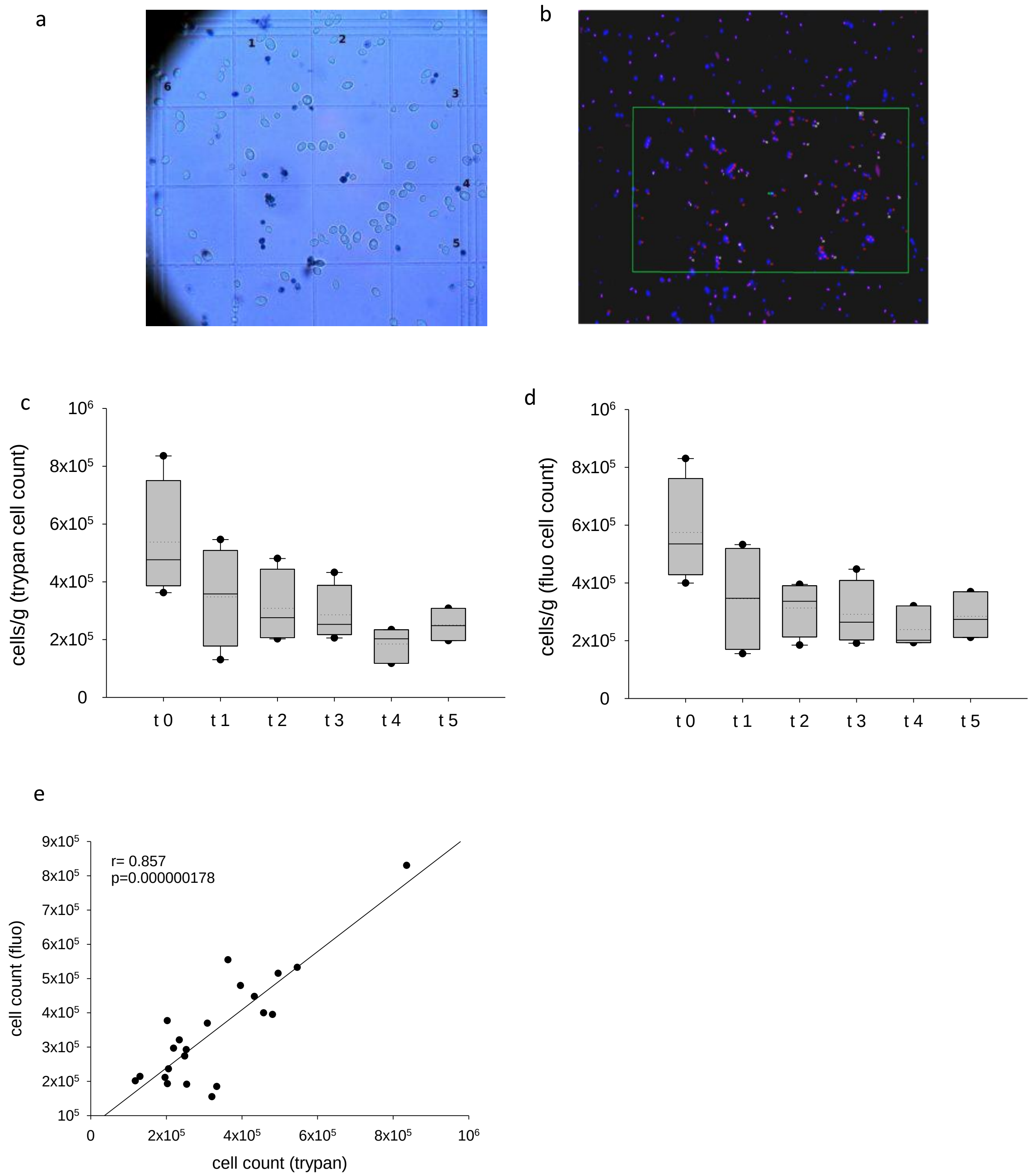

Figure S4. Evaluation of the cell count methods in the SVF (stromal vascular fraction) of fresh (t0) and cryopreserved adipose tissue (t1-t5) by microscopy. **a**) Representative picture of a hemocytometer loaded by cell suspension stained by trypan blue solution. **b**) Representative picture of a hemocytometer loaded by cell suspension stained by ReadyProbes Cell Viability solution. **c**) Number of viable cells normalized by sample weight (g), calculated using trypan blue staining for each time point. **d**) Number of viable cells normalized by sample weight (g), calculated using nuclear fluorescent staining (ReadyProbes) for each time point. e) Correlation between the number of viable cells/g obtained using trypan blue and fluorescence staining.
t0: fresh lipoaspirate, t1: 1 month storage, t2: 2-months storage, t3: 3-months storage, t4: 14-months storage, t5: 36-months storage
